# Retinal Structural and Microvascular Findings During Novel NPR-B-Targeted Vosoritide Therapy in Children with Achondroplasia

**DOI:** 10.3390/jcm15197661

**Published:** 2026-10-03

**Authors:** Anıl Ağaçkesen, Hasan Karakaş, Hande Turan, Elvan Bayramoğlu, Olcay Evliyaoğlu

**Affiliations:** 1 Department of Ophthalmology, Kartal Dr. Lütfi Kırdar Kartal City Hospital, İstanbul 34865, Turkey; 2 Department of Pediatric Endocrinology, Cerrahpaşa Faculty of Medicine, İstanbul University-Cerrahpaşa, İstanbul 34098, Turkey

**Keywords:** achondroplasia, vosoritide, retinal nerve fiber layer, retinal microvasculature

## Abstract

**Background:** The aim of this study was to evaluate the short-term ocular findings during vosoritide therapy in children with achondroplasia using optical coherence tomography (OCT) and OCT angiography (OCTA). **Methods:** In this study, pediatric patients with genetically confirmed achondroplasia underwent standardised ophthalmologic examination, OCT, and OCTA imaging at baseline and after 12 months of vosoritide treatment. Structural parameters, including macular thickness and peripapillary retinal nerve fiber layer (RNFL), as well as vascular densities of the superficial and deep capillary plexuses and choriocapillaris, were analysed. Findings were compared longitudinally and with age- and sex-matched healthy controls. **Results:** A total of 12 patients and 12 controls were included. During the 12-month follow-up, nominal increases were observed in nasal parafoveal thickness and in average, superior, and nasal RNFL thickness (*p* < 0.05); however, none remained significant after false-discovery-rate correction. No significant longitudinal changes were detected in OCTA-derived vascular densities. However, vascular density was significantly lower in specific regions in the achondroplasia group compared to controls both before and after treatment. No clinically relevant ocular pathology was identified. **Conclusions:** No clinically apparent ocular abnormalities or statistically robust longitudinal changes in retinal structure or microvascular perfusion were identified over one year of vosoritide treatment in this small, preliminary cohort.

## 1. Introduction

Achondroplasia is the most common form of skeletal dysplasia, caused by activating mutations in the fibroblast growth factor receptor 3 (FGFR3) gene, with approximately 80% of cases arising de novo [1]. These mutations impair endochondral ossification by inhibiting chondrocyte proliferation and differentiation, leading to disproportionate short stature and characteristic features such as rhizomelic limb shortening, macrocephaly with frontal bossing, midface hypoplasia, and genu varum [2].

Although various ocular manifestations have been reported in association with achondroplasia, the available literature is largely limited to isolated case reports. Reported findings include congenital glaucoma with anterior segment anomalies, chorioretinal coloboma, and cone–rod retinal dystrophy, reflecting the heterogeneous ocular involvement in this condition [3,4,5]. However, these observations are primarily descriptive and based on single cases, without systematic evaluation of retinal structure or microvasculature. To date, no studies have comprehensively assessed ocular findings in patients with achondroplasia using objective imaging modalities such as optical coherence tomography (OCT) and optical coherence tomography angiography (OCTA), nor have cohort-based analyses been performed. This lack of structured imaging data represents a significant gap in the current understanding of ocular involvement in achondroplasia.

Vosoritide is a novel C-type natriuretic peptide (CNP) analogue and a breakthrough therapy as the first approved treatment targeting the molecular pathophysiology of achondroplasia, acting through NPR-B receptor stimulation and enhanced intracellular cGMP signaling to counteract FGFR3 overactivity and promote chondrocyte proliferation and differentiation [6]. Approved by the FDA in 2021, it has demonstrated clinical efficacy with an acceptable safety profile [7]. However, its potential effects on ocular tissues remain unclear.

Given that natriuretic peptide receptors are expressed in ocular tissues, vosoritide may exert direct effects on the eye and influence ocular structure and function [8,9,10]. Based on this, we hypothesized that vosoritide treatment could lead to measurable changes in ocular parameters. Therefore, the aim of this study was to evaluate ocular findings in children with achondroplasia before and after vosoritide therapy, providing an initial clinical assessment of its short-term ocular effects.

## 2. Material and Methods

### 2.1. Study Design and Population

Between 2024 and 2025, pediatric and adolescent patients diagnosed with achondroplasia and initiated on vosoritide therapy in the Pediatric Endocrinology Clinic of a university hospital were retrospectively evaluated using data obtained during routine clinical follow-up. This study protocol was approved by the local ethics committee (approval number: 2025/010.99/21/1; approval date: 30 October 2025) prior to data extraction and statistical analysis. As this was a retrospective study, the control group consisted of healthy children approximately matched for age and sex to the achondroplasia group at baseline, using age at the time of imaging; controls were healthy volunteer children who underwent a single OCT and OCTA imaging session in our clinic and were found to have no ocular pathology on examination.

The diagnosis of achondroplasia was confirmed by characteristic clinical and radiological findings, as well as the identification of the c.1138G>A (p.Gly380Arg) variant in the FGFR3 gene. Prior to initiating vosoritide therapy, written informed consent regarding treatment administration was obtained from the parents of all patients. Vosoritide was administered subcutaneously once daily at approximately 21:00, at a dose of 15 µg/kg, in accordance with the manufacturer’s recommendations. Treatment was initiated in the hospital setting, after which injections were continued at home by trained parents or caregivers. None of the patients had a history of hypertension or were receiving antihypertensive medication, and no hypotensive episodes were documented during the study period; all patients were clinically normotensive. Two patients had radiologically documented foramen magnum stenosis, which was being followed conservatively without surgical intervention; none of the patients had clinical or radiological evidence of hydrocephalus or raised intracranial pressure during the study period.

Before starting treatment, all patients underwent a baseline ophthalmologic examination, including biomicroscopy and fundus examination, and follow-up examinations were performed at the 12th month using the same protocol. Patients and caregivers were also questioned regarding ocular symptoms (e.g., blurred vision, pain, redness) at each visit; no ocular complaints were reported throughout the follow-up period. Refractive error was assessed by autorefractometry as part of the baseline ophthalmologic examination, and spherical and cylindrical values were within ±1.00 diopters in all participants in both groups.

### 2.2. OCT and OCTA Imaging

OCT and OCTA images were obtained using a DRI OCT Triton device (Topcon, Tokyo, Japan). Imaging was performed without pupillary dilation, in the morning (09:00–12:00) to minimize diurnal variation, by the same operator at both visits. Image grading was performed by an observer masked to visit (baseline vs. follow-up) and group (achondroplasia vs. control) assignment. Macular OCT measurements were obtained using the 3D Macula scan protocol; central foveal thickness was assessed within a central circular zone of 1 mm diameter, and parafoveal thickness within the surrounding inner ring (1–3 mm diameter) of the ETDRS grid, divided into nasal, superior, temporal, and inferior quadrants. Retinal thickness values were based on segmentation between the internal limiting membrane and the retinal pigment epithelium. The right eye of each participant was included in the analysis to avoid inter-eye correlation bias. Only OCT and OCTA images with a signal strength index (SSI) greater than 40 were included in the analysis to ensure adequate image quality. Images with visible motion artifacts or segmentation errors were also visually inspected and excluded, in addition to the SSI-based quality threshold.

Peripapillary retinal nerve fiber layer (RNFL) thickness was measured using the 3D Disc scan protocol, with a circular scan with a diameter of 3.45 mm centered on the optic disc. The peripapillary region was defined as a 1.0 mm wide annulus surrounding the optic disc margin, and both global and quadrant-specific RNFL thicknesses were automatically calculated using the device’s segmentation software.

OCTA imaging was performed over a 6 × 6 mm^2^ macular area. Retinal microvascular layers were segmented as follows: the superficial capillary plexus (SCP), extending from 2.6 µm below the internal limiting membrane (ILM) to 15.6 µm below the inner plexiform layer (IPL); the deep capillary plexus (DCP), from 15.6 µm to 70.2 µm below the IPL; and the choriocapillaris, from Bruch’s membrane to 20.2 µm beneath it. Vascular density (%) of each plexus was quantified using the device’s proprietary software (IMAGEnet 6, Topcon).

The foveal avascular zone (FAZ) area was manually measured three times, and the mean value was used for analysis.

### 2.3. Statistical Analysis

Statistical analyses were performed using IBM SPSS Statistics version 23.0 (IBM Corp., Armonk, NY, USA). The distribution of variables was assessed using the Shapiro–Wilk test, and continuous variables were expressed as mean ± standard deviation or median (interquartile range) as appropriate. Due to the small sample size, non-parametric tests were used: the Wilcoxon signed-rank test for paired baseline-to-follow-up comparisons and the Mann–Whitney U test for comparisons with the control group, with effect sizes (rank-biserial correlation) and Hodges–Lehmann estimates (95% CI) calculated to complement *p*-values. As no primary endpoint was prespecified, all parameters were treated as exploratory, and the Benjamini–Hochberg false-discovery-rate procedure was applied within six outcome families (macular thickness, RNFL, SCP, DCP, choriocapillaris, and FAZ) for each comparison type; adjusted q-values are reported alongside nominal *p*-values in Table 1 and Table 2, with *p* < 0.05 considered nominally significant and q < 0.05 considered significant after correction for multiplicity.

## 3. Results

A total of 12 patients with achondroplasia (6 females, 6 males) and 12 age- and sex-matched healthy controls (6 females, 6 males) were included in the study. To avoid inter-eye correlation bias, only one eye per participant was included in the analysis. The mean age was 8.33 ± 1.96 years (range: 4–11 years) in the achondroplasia group and 7.83 ± 2.28 years (range: 4–12 years) in the control group, with no statistically significant difference between groups either at baseline (*p* = 0.410) or at the 12-month examination, when the achondroplasia group would have been approximately 9.33 years old (*p* = 0.081). Best-corrected visual acuity was 1.0 (Snellen) in all participants, and no pathological findings were observed on biomicroscopic examination throughout the follow-up period.

When compared with the control group, vascular density in the SCP was significantly lower in the achondroplasia group at baseline in the nasal (*p* = 0.020), inferior (*p* < 0.001), and temporal (*p* = 0.045) quadrants. After Benjamini–Hochberg false-discovery-rate (FDR) correction, only the inferior quadrant remained statistically significant at baseline (q = 0.001) and at the 12-month examination (*p* = 0.006, q = 0.028), while the nasal and temporal differences did not remain significant after correction.

In macular OCT analysis, a nominal increase was observed in nasal parafoveal thickness at the 12-month examination compared with baseline (*p* = 0.045); however, this did not remain significant after FDR correction (q = 0.188). No significant changes were detected in central foveal thickness or in the superior, inferior, and temporal quadrants (all *p* > 0.05). When compared with the control group, post-treatment measurements showed a nominal difference in the temporal parafoveal quadrant (*p* = 0.020), which did not remain significant after FDR correction (q = 0.102), while all other macular parameters were comparable between groups (all *p* > 0.05).

Regarding peripapillary RNFL measurements, nominal increases were observed in average RNFL thickness (*p* = 0.033), as well as in the superior (*p* = 0.031) and nasal quadrants (*p* = 0.049) at the 12-month examination compared with baseline; however, none remained significant after FDR correction (all q = 0.081). No significant changes were detected in the inferior and temporal quadrants (all *p* > 0.05).

In the DCP, foveal vascular density was significantly lower in the achondroplasia group both before (*p* < 0.001, q = 0.002) and at the 12-month examination (*p* = 0.007, q = 0.034) compared with the control group. No significant differences were observed in other DCP regions. No significant differences were detected in the choriocapillaris layer between groups (all *p* > 0.05).

A detailed summary of OCT parameters is presented in Table 1, and OCTA parameters are summarised in Table 2.

## 4. Discussion

This study provides a comprehensive evaluation of structural and microvascular retinal changes in children with achondroplasia undergoing vosoritide therapy using OCT and OCTA. The main findings indicate nominal increases in RNFL thickness and localised nasal parafoveal thickening during 12 months of vosoritide treatment; however, these findings did not remain significant after FDR correction. Importantly, these changes were not accompanied by any clinical signs of ocular pathology, with no evident ocular abnormalities observed during this preliminary 12-month assessment.

RNFL thickness is a sensitive marker of axonal integrity and can be influenced by multiple factors, including intraocular pressure and developmental processes. Age-related effects should also be considered when interpreting RNFL changes. Although RNFL thickness generally decreases with age in healthy populations, this decline occurs gradually over decades rather than within short time intervals [11]. In pediatric populations, findings are less consistent; some studies report no association with age or axial length, while others suggest a potential increase in RNFL thickness related to ongoing axonal maturation, glial proliferation, or changes in the peripapillary vascular network [12,13]. In addition, the presence of NPR-B receptors in ocular tissues and the known intraocular pressure lowering effects of CNP raise the theoretical possibility of a pharmacological contribution, although receptor expression alone does not establish that systemically administered vosoritide reaches the retina or optic nerve at biologically relevant concentrations. Given the sensitivity of RNFL thickness to intraocular pressure fluctuations, even within physiological ranges, subtle increases could theoretically occur in response to reduced IOP, although IOP was not measured in this study [8,14,15,16,17].

In the present study, nominal increases in average, superior, and nasal RNFL thickness were observed following 12 months of vosoritide treatment; however, none remained significant after FDR correction. Although such findings may raise suspicion for optic disc edema, the absence of clinical or fundoscopic signs argues strongly against true edema. Taken together, the observed increase in RNFL thickness may reflect physiological developmental changes and/or changes occurring during vosoritide treatment, rather than pathological swelling. However, the lack of intraocular pressure measurements represents an important limitation of the study and precludes definitive conclusions regarding the underlying mechanism.

CNP is expressed in retinal tissues and may play a role in retinal physiology, including regulation of the blood–retina barrier and extracellular fluid balance [10]. In the present study, a nominal increase in nasal parafoveal thickness was observed following vosoritide treatment; however, this finding did not remain significant after FDR correction, while other regions remained unchanged. This localised effect suggests the absence of generalised macular thickening. The observed increase may be explained by both physiological and pharmacological mechanisms. In pediatric populations, macular thickness may increase with age due to ongoing structural maturation, while CNP-mediated signaling may additionally influence retinal fluid dynamics and vascular regulation [18].

However, the absence of diffuse or uniform macular thickening suggests that vosoritide does not induce generalised retinal alterations. Therefore, the localised nasal parafoveal thickening may reflect a combination of developmental and changes occurring during vosoritide treatment, although measurement variability cannot be excluded, and does not appear to represent a clinically relevant structural change.

In contrast, vascular density was significantly lower in selected regions in the achondroplasia group compared with age- and sex-matched controls, with inferior SCP and foveal DCP differences remaining significant after FDR correction both before and after treatment, although the post-treatment comparison should be interpreted with some caution given the residual age difference between groups. The persistence of these differences, together with the absence of significant longitudinal vascular changes, suggests that they are unlikely to be treatment-related, although other contributing factors cannot be excluded.

CNP, the active pathway targeted by vosoritide, is a potent vasodilator that plays a key role in endothelial function and vascular tone regulation [19]. In addition, natriuretic peptides have been shown to inhibit vascular smooth muscle proliferation and suppress vascular endothelial growth factor mediated angiogenic signaling [20,21]. Despite these theoretical effects, our findings suggest that systemic vosoritide therapy does not translate into measurable alterations in retinal vascular density over a one-year period.

An important finding of this study is the presence of persistent differences in retinal vascular density between patients with achondroplasia and healthy controls. To our knowledge, this is the first cohort study to comprehensively evaluate ocular findings in patients with achondroplasia using both OCT and OCTA, providing a detailed structural and microvascular assessment.

Overall, no clinically relevant structural or vascular retinal changes were observed during 12 months of vosoritide treatment. Although nominal increases in RNFL thickness and localised macular thickening were observed, these findings did not remain significant after FDR correction and were not accompanied by any clinical signs of optic disc edema or retinal pathology.

Several limitations should be acknowledged. The relatively small sample size may limit statistical power and the generalizability of the findings, and is insufficient to exclude uncommon ocular adverse events or modest structural and vascular effects. The lack of a longitudinal untreated achondroplasia control group should also be considered when interpreting the changes observed during vosoritide treatment. In addition, intraocular pressure and axial length were not measured and formal test–retest reliability of OCT/OCTA measurements was not assessed; DRI OCT Triton measurements are known to exhibit test–retest variability comparable in magnitude to some of the differences observed in this study, which may have influenced the small structural differences observed [22,23]. The 12-month follow-up period further restricts conclusions regarding the long-term ocular course during vosoritide therapy. Taken together, these findings indicate no clinically apparent ocular abnormalities during the 12-month follow-up period. The findings of this study should be interpreted as exploratory and hypothesis-generating, and require confirmation in larger, prospective studies.

## Figures and Tables

**Table 1:** Optical coherence tomography parameters before and after vosoritide treatment.

Parameter	First Examination (x)	After Vosoritide Treatment (y)	Control Group (z)	*p* and q (x − y)	Effect Size r; Δ [95% CI] (x − y)	*p* and q (x − z)	Effect Size r; Δ [95% CI] (x − z)	*p* and q (y − z)	Effect Size r; Δ [95% CI] (y − z)
**Macular OCT (µm)**									
Foveal	238.41 ± 28.08	245.91 ± 34.39	245.33 ± 20.47	0.432 0.432	r = −0.26 Δ = −1.75 [−18.50, 5.00]	0.590 0.671	r = −0.14 Δ = −9.00 [−27.00, 19.00]	0.799 0.998	r = −0.07 Δ = −3.50 [−25.00, 25.00]
Superior	308 (296.25–328)	315.82 ± 22.04	305.41 ± 15.69	0.130 0.217	r = −0.52 Δ = −4.00 [−25.00, 1.00]	0.671 0.671	r = 0.12 Δ = 3.00 [−13.00, 20.00]	0.291 0.486	r = 0.26 Δ = 8.00 [−9.00, 29.00]
Nasal	310 ± 16.27	318.83 ± 19.73	300.75 ± 19.68	**0.045** 0.188	r = −0.65 Δ = −8.00 [−16.50, 0.00]	0.410 0.671	r = 0.21 Δ = 7.00 [−9.00, 25.00]	0.101 0.251	r = 0.40 Δ = 17.50 [−2.00, 34.00]
Inferior	298 (283.5–309.75)	305.58 ± 20.80	302.08 ± 15.71	0.239 0.299	r = −0.39 Δ = −11.50 [−36.00, 4.50]	0.443 0.671	r = −0.19 Δ = −6.00 [−24.00, 14.00]	1.000 1.000	r = 0.00 Δ = 0.00 [−13.00, 18.00]
Temporal	296.5 (286.5–313)	310 ± 20.39	289.58 ± 17.27	0.075 0.188	r = −0.61 Δ = −13.50 [−35.00, 1.50]	0.347 0.671	r = 0.24 Δ = 8.00 [−10.00, 23.00]	**0.020** 0.102	r = 0.56 Δ = 19.00 [2.00, 37.00]
**Peripapillary RNFL (µm)**									
Average	106.5 ± 10.1	108.75 ± 11.59	106.75 ± 8.37	**0.033** 0.081	r = −0.69 Δ = −2.00 [−4.00, 0.00]	0.799 0.977	r = −0.08 Δ = −1.00 [−9.00, 8.00]	0.799 0.843	r = 0.08 Δ = 1.00 [−8.00, 11.00]
Superior	137 ± 16.34	141.91 ± 13.78	136.75 ± 15.08	**0.031** 0.081	r = −0.70 Δ = −4.25 [−9.00, −0.50]	0.977 0.977	r = 0.01 Δ = 1.00 [−14.00, 15.00]	0.590 0.843	r = 0.14 Δ = 5.00 [−9.00, 17.00]
Nasal	81.91 ± 11.57	85.33 ± 13.47	82 (78.75–87)	**0.049** 0.081	r = −0.64 Δ = −3.00 [−7.00, 0.00]	0.799 0.977	r = −0.08 Δ = −1.00 [−12.00, 8.00]	0.843 0.843	r = 0.06 Δ = 1.00 [−10.00, 12.00]
Inferior	135.25 ± 20.44	135.75 ± 20.92	130.08 ± 13.17	0.969 0.969	r = 0.01 Δ = 0.00 [−3.50, 2.00]	0.630 0.977	r = 0.13 Δ = 3.00 [−10.00, 17.00]	0.755 0.843	r = 0.09 Δ = 2.50 [−10.00, 20.00]
Temporal	72.5 ± 10.36	70.83 ± 8.2	75.83 ± 13.75	0.222 0.278	r = 0.40 Δ = 1.50 [−1.50, 4.50]	0.671 0.977	r = −0.11 Δ = −2.00 [−15.00, 7.00]	0.551 0.843	r = −0.16 Δ = −3.00 [−15.00, 6.00]

x = baseline; y = 12-month examination (achondroplasia group); z = healthy control group. Wilcoxon signed-rank test (x − y); Mann–Whitney U test (x − z, y − z). r = rank-biserial correlation; Δ = Hodges–Lehmann estimate with 95% CI. Values shown are nominal *p* followed by Benjamini–Hochberg-adjusted q, calculated within each outcome family (Macular, RNFL, SCP, DCP, CC, FAZ). Bold indicates *p* < 0.05 or q < 0.05.

**Table 2:** Optical coherence tomography angiography parameters before and after vosoritide treatment.

Parameter	First Examination (x)	After Vosoritide Treatment (y)	Control Group (z)	*p* and q (x − y)	Effect Size r; Δ [95% CI] (x − y)	*p* and q (x − z)	Effect Size r; Δ [95% CI] (x − z)	*p* and q (y − z)	Effect Size r; Δ [95% CI] (y − z)
**Superficial capillary plexus density (%)**									
Foveal	21.41 ± 4.56	21.79 ± 5.73	22.16 ± 4.03	0.638 0.754	r = −0.15 Δ = −0.47 [−2.14, 1.03]	0.478 0.478	r = −0.18 Δ = −1.59 [−4.69, 2.72]	0.514 0.514	r = −0.17 Δ = −0.99 [−4.88, 3.28]
Superior	42.03 ± 4.38	42.54 ± 4.53	44.75 ± 3.21	0.530 0.754	r = −0.20 Δ = −1.00 [−4.67, 3.53]	0.114 0.142	r = −0.39 Δ = −2.69 [−5.95, 0.51]	0.266 0.434	r = −0.28 Δ = −1.94 [−6.07, 1.69]
Nasal	42.05 ± 3.57	42.63 ± 5.6	44.98 ± 2.05	0.695 0.754	r = −0.13 Δ = −0.63 [−4.03, 2.74]	**0.020** 0.051	r = −0.56 Δ = −2.62 [−6.10, −0.29]	0.347 0.434	r = −0.24 Δ = −1.32 [−7.25, 2.66]
Inferior	37.1 ± 3.8	38.2 ± 4.25	42.52 ± 1.97	0.084 0.422	r = −0.56 Δ = −0.89 [−2.34, 0.06]	**0.0002** **0.001**	r = −0.83 Δ = −5.22 [−8.46, −2.38]	**0.006** **0.028**	r = −0.65 Δ = −4.63 [−7.18, −1.17]
Temporal	42.38 ± 4.14	42.53 ± 5.05	46.3 ± 2.92	0.754 0.754	r = 0.10 Δ = 0.39 [−2.23, 1.70]	**0.045** 0.075	r = −0.49 Δ = −3.48 [−7.38, −0.05]	0.068 0.171	r = −0.44 Δ = −3.04 [−6.90, 0.48]
**Deep capillary plexus density (%)**									
Foveal	19.46 ± 6.19	18.93 (18.13–21.78)	28.39 ± 3.27	0.084 0.422	r = −0.56 Δ = −1.52 [−3.75, 0.67]	**0.0005** **0.002**	r = −0.79 Δ = −9.03 [−14.12, −4.08]	**0.007** **0.034**	r = −0.64 Δ = −9.18 [−12.28, −3.26]
Superior	43.62 ± 4.8	42.86 ± 5.36	40.24 ± 3.63	0.937 0.937	r = 0.03 Δ = 0.17 [−2.16, 3.93]	0.128 0.213	r = 0.38 Δ = 2.94 [−0.79, 7.11]	0.089 0.222	r = 0.42 Δ = 2.81 [−0.67, 6.81]
Nasal	41.61 ± 3.7	41.96 ± 6.23	41.84 ± 2.69	0.308 0.513	r = −0.33 Δ = −0.81 [−2.38, 1.43]	0.887 0.887	r = 0.04 Δ = 0.34 [−2.84, 2.74]	0.514 0.642	r = 0.17 Δ = 1.43 [−2.58, 4.60]
Inferior	38.94 ± 3.55	36.71 ± 6.33	36.49 ± 2.62	0.695 0.869	r = 0.13 Δ = 0.98 [−2.56, 7.12]	0.052 0.130	r = 0.47 Δ = 2.71 [−0.07, 5.12]	0.378 0.629	r = 0.22 Δ = 1.52 [−3.29, 4.72]
Temporal	42.43 ± 3.91	40.16 ± 6.21	40.33 ± 4.28	0.239 0.513	r = 0.39 Δ = 1.25 [−1.33, 7.09]	0.266 0.332	r = 0.28 Δ = 1.83 [−1.63, 5.62]	0.932 0.932	r = 0.04 Δ = 0.23 [−4.59, 4.91]
**Choriocapillaris density (%)**									
Foveal	54.63 (52.33–55.89)	53.55 ± 8.69	53.92 ± 4.25	0.638 0.638	r = −0.15 Δ = −0.38 [−1.93, 1.92]	0.799 0.887	r = 0.07 Δ = 0.42 [−3.93, 4.28]	0.713 0.713	r = 0.10 Δ = 1.11 [−4.68, 5.90]
Superior	52.84 ± 2.33	50.5 ± 4.11	53.64 ± 4.66	0.117 0.292	r = 0.51 Δ = 1.63 [−0.32, 5.74]	0.514 0.887	r = −0.17 Δ = −0.89 [−4.30, 2.66]	0.128 0.638	r = −0.38 Δ = −2.87 [−7.00, 0.89]
Nasal	51.97 ± 2.14	52.8 ± 2.87	51.77 ± 4.36	0.272 0.453	r = −0.36 Δ = −0.86 [−2.79, 0.98]	0.887 0.887	r = 0.04 Δ = 0.42 [−3.08, 3.52]	0.378 0.713	r = 0.22 Δ = 1.03 [−2.14, 4.78]
Inferior	50.32 ± 4.42	48.29 ± 4.56	50.04 ± 5.02	0.099 0.292	r = 0.54 Δ = 2.23 [−0.40, 4.70]	0.630 0.887	r = 0.13 Δ = 0.91 [−3.76, 4.59]	0.590 0.713	r = −0.14 Δ = −1.13 [−5.11, 2.41]
Temporal	52.52 ± 4.26	52.69 ± 4.76	51.48 ± 3.18	0.583 0.638	r = −0.18 Δ = −0.30 [−2.18, 1.64]	0.551 0.887	r = 0.15 Δ = 1.05 [−2.39, 4.50]	0.551 0.713	r = 0.15 Δ = 0.98 [−2.28, 4.62]
**Foveal avascular zone (µm^2^)**									
Superficial	298.53 ± 46.25	294.08 ± 64.72	327.56 ± 99.45	0.937 0.937	r = −0.03 Δ = −0.36 [−14.93, 24.73]	0.843 0.843	r = −0.06 Δ = −16.15 [−111.09, 47.81]	0.630 0.630	r = −0.13 Δ = −25.66 [−125.00, 57.30]
Deep	319.5 ± 64.18	312.73 ± 77.85	364.51 ± 70.07	0.272 0.544	r = 0.36 Δ = 11.79 [−10.92, 46.75]	0.128 0.255	r = −0.38 Δ = −43.78 [−106.17, 11.95]	0.160 0.320	r = −0.35 Δ = −54.79 [−118.48, 18.44]

x = baseline; y = 12-month examination (achondroplasia group); z = healthy control group. Wilcoxon signed-rank test (x − y); Mann–Whitney U test (x − z, y − z). r = rank-biserial correlation; Δ = Hodges–Lehmann estimate with 95% CI. Values shown are nominal *p* followed by Benjamini–Hochberg-adjusted q, calculated within each outcome family (Macular, RNFL, SCP, DCP, CC, FAZ). Bold indicates *p* < 0.05 or q < 0.05.

## Data Availability

The data supporting the findings of this study are available from the corresponding author upon reasonable request.
